# The Great Game between Plants and Viruses: A Focus on Protein Homeostasis

**DOI:** 10.3390/ijms241612582

**Published:** 2023-08-09

**Authors:** Hangjun Sun, Xinxin Jing, Chaonan Wang, Pengyue Wang, Ziting Huang, Bingjian Sun, Pengbai Li, Honglian Li, Chao Zhang

**Affiliations:** 1 The Engineering Research Center for Plant Health Protection Technology in Henan Province, Henan Agricultural University, Zhengzhou 450002, China; 2 Department of Plant Pathology, College of Plant Protection, Henan Agricultural University, Zhengzhou 450002, China

**Keywords:** protein homeostasis, protein stability, ubiquitin-proteasome degradation, autophagy

## Abstract

Plant viruses are tiny pathogenic obligate parasites that cause significant damage to global crop production. They exploit and manipulate the cellular components of host plants to ensure their own survival. In response, plants activate multiple defense signaling pathways, such as gene silencing and plant hormone signaling, to hinder virus propagation. Growing evidence suggests that the regulation of protein homeostasis plays a vital role in the ongoing battle between plants and viruses. The ubiquitin-proteasome-degradation system (UPS) and autophagy, as two major protein-degradation pathways, are widely utilized by plants and viruses in their arms race. One the one hand, these pathways act as essential components of plant’s antiviral defense system by facilitating the degradation of viral proteins; on the other hand, viruses exploit the UPS and autophagy to create a favorable intracellular environment for viral infection. This review aims to provide a comprehensive summary of the events involved in protein homeostasis regulation during viral infection in plants. Gaining knowledge in this area will enhance our understanding of the complex interplay between plants and viruses.

## 1. Introduction

Plants, as immobile organisms, are frequently attacked by a number of pathogens (parasites), including fungi, bacteria, parasitic seed plants, nematodes, and viruses. These pathogens pose a significant threat to global or local agricultural production. Unlike other pathogenic organisms, viruses lack a cellular structure and can only parasitize within host cells. Besides, most viruses are transmitted by insect vectors including aphids, planthoppers, leafhoppers, etc. Therefore, classical immune mechanisms in plants, such as the secretion of secondary metabolites to kill pathogen cells or the reinforcement of cell walls and epidermal structures to block pathogen invasion, often prove ineffective against viruses [1]. However, throughout the course of their long-term co-evolution with viruses, plants have developed sophisticated and distinct antiviral immune pathways. These include RNA interference, a dominant pathway directly targeting the viral nucleic acids for gene silencing, as well as various mechanisms for regulating protein homeostasis [2,3]. Key regulatory processes involved in maintaining protein homeostasis during viral infections include the ubiquitin-proteasome-degradation system (UPS) and autophagy [4,5,6,7].

The degradation of a protein via the UPS proceeds in two discrete and successive steps: covalent attachment of multiple ubiquitin (Ub) molecules, a 76-residue protein that is highly conserved throughout the eukaryotic kingdom, to the protein substrates (referred to as ubiquitination or ubiquitylation) and degradation of the targeted proteins by the 26S proteasome complex. Three classes of enzymes sequentially regulate the ubiquitination process: (1) Ub-activating enzymes (E1) activate Ub and transfer it onto the E1 enzyme; (2) Ub-conjugating enzymes (E2) receive Ub from the E1 enzyme; (3) various types of E3 ligases transfer Ub from the E2 enzyme to the protein substrate. E3 ligases, which form a large protein family, determine the specificity of substrate ubiquitination [8]. To ensure the dynamic balance of cellular proteins during plant growth and development or response to various stresses, ubiquitination can be reversed by enzymes called Ub hydrolases or deubiquitinating enzymes (DUBs) [9]. Most of these enzymes are Cys proteinases that cleave isopeptidase bonds. They either trim poly-Ub chains or remove them from substrate proteins, thus contributing to the reversal of signaling events through regulating protein stabilization. The involvement of the UPS in plant–virus interactions is increasingly evident. In some cases, viral proteins themselves become targets of Ub conjugation events, while in others, viral proteins exploit the UPS to create a favorable intracellular environment for viral infection [6,10].

Autophagy, derived from the Greek word meaning “self-eating”, is an evolutionarily conserved degradation pathway that targets macromolecules, organelles, and pathogens [11]. Plants have three types of autophagy: macroautophagy (referred to as autophagy), microautophagy, and mega-autophagy [12]. Macroautophagy, commonly known as autophagy, represents the extensively studied autophagy pathway in plants. Depending on the cargo specificity, autophagy can be further divided into two types: bulk autophagy and selective autophagy. During the process of autophagy, double-membrane-bound vesicles called autophagosomes form through a series of steps and then fuse with host cell vacuoles (in yeast and plants) or lysosomes (in mammals), thereby facilitating the proteolytic degradation and recycling of cargo molecules. Plant autophagy involves more than 40 autophagy-related genes (ATGs) that play vital roles in phagophore initiation, phagophore nucleation, autophagosome expansion, and vacuolar membrane fusion. Recent studies have shed light on the dual role of autophagy in viral infection, emphasizing its intricate involvement in regulating plant–virus interactions [13]. One the one hand, autophagy serves as an integral part of plant’s antiviral defense system by facilitating the degradation of viral proteins; on the other hand, viruses exploit autophagy for their replication, underscoring the complex interplay between autophagy and viral dynamics [14].

This review aims to comprehensively summarize the events associated with regulating protein homeostasis during viral infection in plants, with a particular focus on the roles of the UPS and autophagy. Gaining knowledge in this area will enhance our understanding of the interplay between plants and viruses.

## 2. Friend or Foe: The Dual Role of UPS in Plant Virus Infection

**Figure 1:**
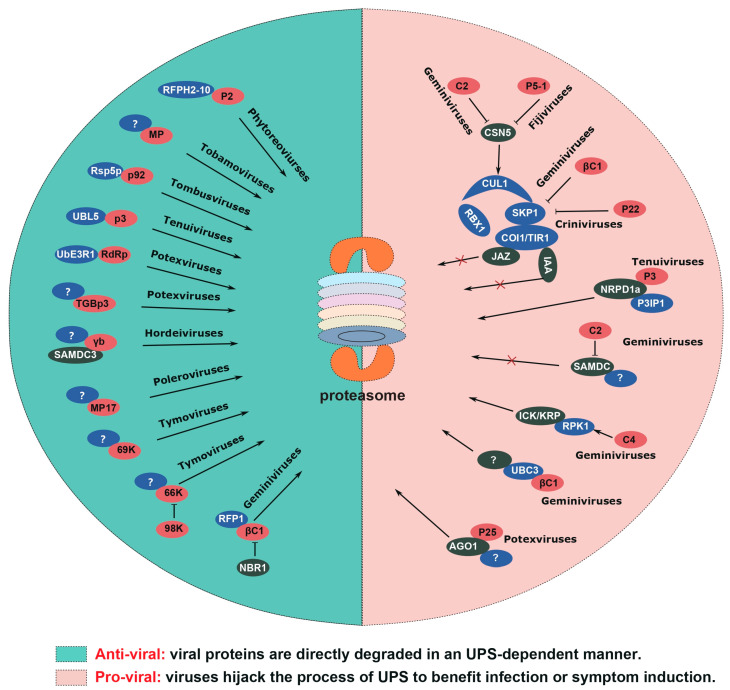
**The dual role of the UPS in plant virus infection.** The left section, distinguished by a green background, elucidates the antiviral function of the UPS. In these cases, virus-encoded proteins are degraded by the 26S proteasome and are indicated in red. Ubiquitin-related proteins responsible for targeting viral proteins are marked in blue, while other host proteins are marked in green. The right section, distinguished by a red background, illustrates the involvement of the UPS as a virulence factor in viral replication and symptom formation. Viruses manipulate the UPS to either promote or inhibit the degradation of specific plant proteins, thereby facilitating viral infection or inducing symptom formation.

The UPS is a highly conserved mechanism that plays a crucial role in finely regulating protein homeostasis across various cellular processes [15]. Early studies revealed the altered expression profiles of UPS-associated genes during viral infections. For example, in tobacco, infection by tobacco mosaic virus (TMV) induced the transcription of genes encoding the ubiquitin-activating enzymes (E1), namely NtE1A and NtE1B [16]. Besides, the manipulation of certain UPS-related genes will have an impact on the resistance of host plants against viruses [17,18,19,20]. However, the direct evidence of the UPS’s involvement in plant–virus interplays was lacking at that time. With the advancement of molecular biological technologies and the establishment of in vitro ubiquitination experimental systems, mounting evidence suggests that the UPS plays a significant role in plant–virus interactions [21]. It is now evident that the UPS acts as a double-edged sword during viral pathogenesis, alternatively impairing and facilitating viral infection (Figure 1).

### 2.1. UPS Directly Targets Viral Proteins for Degradation

The ubiquitination of viral protein was initially observed in the case of TMV, where viral particles underwent ubiquitination, specifically at lysine 53 in the coat protein (CP) [22]. Furthermore, the TMV movement protein (MP) was shown to be degraded by the 26S proteasome [23,24]. A single amino acid change at the R3 site of TMV MP allows the evasion of UPS-dependent degradation, thereby enhancing its viral transport function [24]. Subsequent research has demonstrated that ubiquitination of viral proteins is a widespread occurrence and involves various viral components such as MPs [25,26,27], RNA-dependent RNA polymerases (RdRps) [28], CPs [29], and viral suppressors of RNA silencing (VSRs) from various viruses.

Moreover, the corresponding components of the UPS responsible for the degradation of viral proteins have been identified. For example, the Nedd4 family E3 ubiquitin ligase Rsp5p inhibits tomato bushy stunt virus (TBSV) replication by regulating the degradation of the p92 (RdRp) [30,31,32]. An E3 ubiquitin ligase containing a really interesting new gene (RING) domain 1 (named NbUbE3R1) targets the RdRp of bamboo mosaic virus (BaMV) and restricts its replication [33]. OsRFPH2-10, a RING-H2 finger E3 ubiquitin ligase, plays a role in rice antiviral defense during the early stages of rice dwarf virus (RDV) infection by targeting P2 (CP) for degradation [29]. The ubiquitin-like protein 5 (NbUBL5) interacts with P3, a VSR of rice stripe virus (RSV), and mediates its degradation through the 26S proteasome pathway [34]. In some cases, viral proteins are targeted for degradation by plant “adaptor proteins” that recruit them to the UPS. For instance, S-adenosylmethionine decarboxylase 3 (SAMDC3) interacts with barley stripe mosaic virus (BSMV) γb protein (VSR) and promotes its proteasomal degradation by increasing its ubiquitination [35].

### 2.2. Viruses Escape from UPS-Mediated Degradation of Viral Proteins

To evade host UPS-mediated antiviral responses, certain viruses enlist host proteins to shield themselves from degradation. Shen et al. (2016) reported that the RING-finger protein NbRFP1 interacts with the tomato yellow leaf curl China betasatellite (TYLCCNB) βC1 protein and targets it for degradation through the UPS [36]. In response, βC1 recruits NEIGHBOR OF BREAST CANCER 1 (NbNBR1), a host autophagic receptor, forming cytoplasmic granules that protect itself from NtRFP1-mediated degradation [37]. Additionally, the small ubiquitin-related modifier (SUMO) attached to βC1 of synedrella yellow vein clearing virus (SyYVCV), a newly identified member of the Begomovirus genus of the geminiviruses, appears to maintain its protein stability by preventing UPS-mediated degradation [38]. Li et al. (2019) reported that the silencing suppressor P0 from brassica yellows virus (BrYV) interacts with SKP1. Intriguingly, this interaction safeguards P0 from degradation by the proteasome and autophagy pathways [39]. Moreover, some viruses even encode functional proteins with deubiquitinating enzyme activity to protect them from degradation. Camborde et al. (2010) identified a PEST sequence within turnip yellow mosaic virus (TYMV) 66K (RdRp), responsible for its degradation by the UPS [28]. Subsequent studies revealed that TYMV 98K mediates in vivo deubiquitylation of the TYMV 66K protein, leading to its stabilization and, thus, contributes to viral replication [40]. These studies demonstrated that UPS-mediated viral degradation is a common strategy employed by plants to suppress virus infection. In response, viruses have evolved diverse strategies to evade UPS-mediated degradation, reflecting an ongoing arms race between plants and viruses. As research continues to advance, it is anticipated that additional viral proteins regulated by the UPS will be discovered. This ongoing investigation will contribute to expanding our understanding of viral protein homeostasis.

### 2.3. Viruses Hijack UPS for Targeting Host Factors Involved in Antiviral Responses

As one of the most-prevalent post-translational modifications (PTMs), ubiquitylation participates in nearly all physiological and signaling processes in plants, including hormone perception, photomorphogenesis, and circadian rhythms [41]. Besides its well-established antiviral role, the UPS is frequently manipulated by viruses to create a favorable intracellular environment for viral infection. In particular, the SKP1/CUL1/F-box (SCF) complex, a well-studied multi-subunit ubiquitin ligase, regulates several plant-hormone-signaling pathways, such as jasmonates, auxin, and gibberellins, which are crucial for plant development and response to pathogens [42]. However, multiple viruses employ diverse strategies to target the SCF complex and disrupt defense-associated hormone signaling, thus facilitating their replication. For instance, the geminivirus C2 protein and rice black-streaked dwarf virus (RBSDV) P5-1 protein interact with COP9 signalosome 5 (CSN5), hindering CSN-mediated derubylation of SCF complex in Arabidopsis and rice. This interference of SCF function leads to suppressed jasmonate signaling to favor virus infection [43,44]. Furthermore, the cotton leaf curl Multan betasatellite (CLCuMuB) βC1 protein disrupts the integrity of the SCF complex by binding to SKP1, resulting in suppressed jasmonate signaling to help the viral infection [45]. Similarly, tomato chlorosis virus (ToCV) P22 protein interacts with SKP1 and hampers the assembly of the SCF complex, leading to suppressed auxin signaling and facilitating viral infection [46]. In addition to the SCF complexes, viruses also modulate other UPS components or their target proteins. For instance, the beet severe curly top virus (BSCTV) C4 protein induces the transcription of related to KPC1 (RPK, a RING finger E3 ligase), leading to altered host cell cycle progression, which benefits viral infection [47]. The P25 (VSR) of potato virus X (PVX) interacts with Argonaute1 (AGO1) and mediates its degradation through the 26S proteasome [48], thereby suppressing the antiviral RNA silencing and accelerating virus infection. Additionally, the geminivirus C2 protein attenuates UPS-mediated degradation of S-adenosyl-methionine decarboxylase 1 (SAMDC1), resulting in suppressed geminivirus DNA methylation and enhanced viral replication [49]. These findings highlight how viruses strategically hijack the UPS to target key host factors involved in antiviral responses.

### 2.4. Viruses Induce Disease Symptoms by Exploiting UPS

Due to the vital role of the UPS in plant growth and development, the manipulation of the UPS or its target proteins by viruses can lead to an abnormal tissue morphology, which is associated with virus-induced disease symptoms. Our previous study demonstrated that the P3 protein encoded by rice grassy stunt virus (RGSV) serves as a pathogenicity determinant. Constitutive overexpression of P3 in rice results in a series of growth defects consistent with disease symptoms such as stuntedness and excessive tillering. P3 has the ability to target NUCLEAR RNA POLYMERASE D1a (OsNRPD1a), a subunit of plant-specific RNA polymerase IV, for UPS-mediated degradation through P3-inducible protein 1 (named P3IP1), a functional U-box type E3 ubiquitin ligase. Overexpression of P3IP1 or knockdown of OsNRPD1 in rice causes severe stunting and increased tiller numbers, reminiscent of the disease symptoms caused by RGSV infection and P3 overexpression [50]. Another example involves the CLCuMuB βC1 protein, which interacts with SlUBC3 (a tomato ubiquitin-conjugating enzyme), leading to a global reduction of polyubiquitinated protein levels and possibly inducing viral disease symptoms [51]. These studies highlight the significant role of the UPS in viral pathogenesis.

### 2.5. The Role of Cell-Division-Cycle 48 in Maintaining Host and Viral Protein Homeostasis

The UPS plays a crucial role in maintaining protein quality control within the endoplasmic reticulum (ER) through ER-associated degradation (ERAD) [52]. A conserved component of ERAD is p97/cell-division-cycle-48 (CDC48) ATPase, which operates on the cytoplasmic surface of the ER and facilitates the extraction of ubiquitinated proteins from the retrotranslocon for proteasomal degradation [53]. At present, this is the only literature to reveal that CDC48-dependent protein homeostasis regulation is also double-sided in the plant–virus interactions. For example, TBSV utilizes this function of p97/CDC48 to enhance replicase assembly and activity during viral infection [54]. Conversely, in the case of TMV, p97/CDC48 may act as a host-defense mechanism by recognizing viral MP as misfolded and promoting its targeting for degradation, thereby hindering its transit through the ER transport pathway [55,56].

## 3. Autophagy–Virus Interplay in Plants: From Antiviral Recognition to Proviral Manipulation

**Figure 2:**
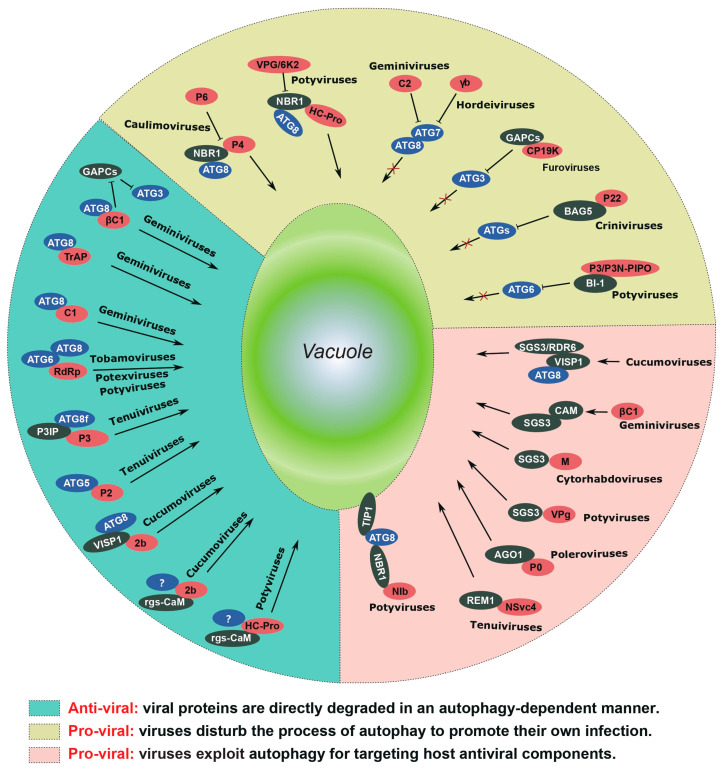
**The dual role of autophagy in plant virus infection.** The left section, characterized by a green background, elucidates the antiviral role of autophagy. In these instances, virus-encoded proteins undergo degradation through autophagy, as indicated by the red mark. Autophagy-related proteins, responsible for targeting viral proteins, are marked in blue, while other host proteins are highlighted in green. The upper section, distinguished by a yellow background, illustrates how viruses hinder cellular autophagy through diverse mechanisms. The bottom right section, featuring a red background, demonstrates how viruses exploit the cellular autophagy pathway for the degradation of immune-related proteins, as well as for virus replication and virion accumulation.

Autophagy serves as a degradation pathway responsible for recycling various cellular components, ranging from cytosolic proteins to entire organelles. The turnover of proteins through autophagy is a vital process essential for the survival of all organisms. In a significant milestone, Liu et al. (2005) reported the first observation of virus-induced autophagy in plants [57]. Using the fluorescent dye LysoTracker Red, they detected autolysosomes in tobacco cells infected by TMV. In *N*-gene-containing *Nicotiana benthamiana* plants, the mRNA and protein levels of Beclin1 increased during the early stages of TMV-induced hypersensitive response (HR), which is associated with programmed cell death (PCD). Knocking down key autophagy-related genes, including ATG3, Beclin1 (ATG6), and ATG7, through virus-induced gene silencing (VIGS) resulted in unrestricted HR PCD and enhanced TMV accumulation [57]. These findings provide compelling evidence for the integration of autophagy into plant immunity. Over the last decade, extensive research has illuminated the intricate involvement of autophagy in the interaction between plants and viruses (Figure 2). Notably, autophagy-related proteins play a dual role in this context. On the one hand, they directly target multiple viral proteins for degradation, serving as a defense mechanism against viral infection. This is evidenced by the promotion of viral infection in autophagy-deficient mutants. On the other hand, viruses manipulate autophagy to evade plant immune defenses and promote their replication. Additionally, virus-induced autophagy can prevent excessive senescence and tissue death in infected plants, thereby significantly prolonging the duration of viral production [58].

### 3.1. Viral Proteins Are Directly Targeted for Degradation through Autophagy

The degradation of viral proteins by plant autophagy was initially discovered by Nakahara et al. Their research revealed that tobacco rgs-CaM, a calmodulin-like protein, binds to the dsRNA-binding domains of several VSRs and stimulates their degradation. The application of 3-methyladenine (3-MA), an autophagy inhibitor, resulted in reduced degradation of VSRs and rgs-CaM. Furthermore, they observed the colocalization of VSRs and rgs-CaM with LysoTracker-stained bodies, markers of autolysosomes [59]. These findings integrate the role of the autophagy-mediated degradation of viral proteins into the plant defense system, establishing it as a countermeasure against viral infection. Recent research has further elucidated the selective autophagy process involved in the degradation of various viral proteins, particularly viral RdRps, CPs, and VSRs [60]. Selective autophagy facilitates the degradation of specific cellular components, relying primarily on diverse autophagic receptors responsible for sequestering specific cargoes into autophagosomes. These autophagic receptors typically contain an ATG8-interacting motif (AIM) or ubiquitin-interacting motif (UIM), which, respectively, bind to the LIR/AIM docking site (LDS) or the UIM docking site (UDS) on ATG8. Thus, ATG8 acts as a docking platform on the autophagosomes membrane [60].

However, the mechanism of targeting viral proteins for autophagic degradation is not always consistent. In some cases, autophagic receptors are involved, while in others, certain ATGs, which are not typical autophagic receptors, assume responsibility for targeting. For instance, a common autophagy cargo receptor called NBR1 has been found to interact with the CP from cauliflower mosaic virus (CaMV), and the helper-component proteinase (HC-Pro), a VSR from turnip mosaic virus (TuMV), leading to autophagic degradation and the suppression of viral accumulation [61,62]. Similarly, a novel selective autophagy cargo receptor, P3 interacting protein in *N. benthamiana* (NbP3IP), was reported to target the P3 protein of RSV for autophagic degradation by interacting with NbATG8f [63]. Another example involves Beclin1, which has recently been identified as a selective autophagy receptor due to the presence of its AIM. Beclin1 directly interacts with TuMV NIb (RdRp) by recognizing its highly conserved GDD motif and interacts with the ATG8a through its AIM. Mutating the AIM in Beclin1 disrupts its interaction with ATG8a, resulting in impaired capacity to induce autophagosome formation and compromised ability to degrade NIb [64]. Recent research reported that the VIRUS-INDUCED SMALL PEPTIDE 1 (VISP1), a selective autophagy receptor containing an ATG8-interacting UIM domain, induces symptom recovery from severe infections of plant viruses through controlling the stability of multiple VSRs, including the well-documented 2b encoded by cucumber mosaic virus (CMV) and the C2/AC2 of two geminiviruses [65].

In some cases, ATGs directly target viral proteins for autophagic degradation. Zhang et al. (2023) reported that ATG5 interacts with the RSV P2 protein and targets it for autophagic degradation [66]. Li et al. (2020) revealed that ATG8h directly interacts with C1 of tomato leaf curl Yunnan virus (TLCYnV) by recognizing its potential AIM. However, TLCYnV carrying the AIM mutation displays enhanced pathogenicity in solanaceous plants, owing to this AIM mutation protecting the viral protein from autophagic degradation [67]. In addition, ATG8 also directly interacts with the βC1 of CLCuMuB and the TrAP of tomato leaf curl New Delhi virus (ToLCNDV), guiding them to autophagosomes for degradation [68,69]. Further studies revealed that βC1 induces autophagy by disrupting the interaction of ATG3 with glyceraldehyde-3-phosphate dehydrogenase (GAPC), a negative regulator of plant autophagy. The mutant virus carrying βC13A, which exhibited reduced capacity to interact with GAPCs and induce autophagy, showed increased symptoms and viral DNA accumulation, underscoring the antiviral role of autophagy [70].

### 3.2. Viral Proteins Interfere with the Autophagy-Mediated Antiviral Responses

To evade autophagy-mediated antiviral signaling, viruses employ various counteractive strategies. Firstly, viral proteins can disturb the function of ATGs through direct interactions, thereby inhibiting the autophagy process. For example, the γb protein from BSMV and C2 from geminiviruses directly interact with ATG7, competitively interfering with the interaction between ATG7 and ATG8, thus subverting autophagy to promote viral infection [71,72]. In a study by Niu et al. (2022), it was discovered that the 19 kDa coat protein (CP19K) of Chinese wheat mosaic virus (CWMV) interacts with cytosolic GAPC and ATG3, potentially leading to the formation of a CP19K-GAPC-ATG3 complex. This complex reduces antiviral autophagic activities, thereby promoting virus infection [73]. Secondly, viruses can inhibit autophagy by downregulating the transcription of ATGs. One such example is observed in the case of tomato chlorosis virus (ToCV) infection. The P22 protein of ToCV interacts with the *N. benthamiana* B-cell lymphoma2-associated athanogenes5 (NbBAG5), an evolutionarily conserved protein mainly involved in plant growth and stress response, and induces NbBAG5 expression, leading to the inhibition of autophagy and favoring viral infection [74]. Another report revealed that the interaction of P3 and/or P3N-PIPO of PVY with BI-1 decreases the expression of the ATG6, thereby inhibiting the autophagic degradation of viral NIb and enhancing viral replication [75]. Thirdly, viruses can induce specific structures to prevent viral protein association with autophagosomes. Hafrén et al. (2017) reported that CaMV P6 induced viral inclusion bodies that antagonized NBR1-mediated targeting of viral capsid protein and particles for autophagic degradation [61]. In the case of TuMV, it was suggested that viral VPg and 6K2 antagonized NBR1-mediated autophagic degradation of HC-Pro, through unknown mechanisms [62].

### 3.3. Viruses Exploit the Autophagy to Suppress Antiviral Signaling

Despite its antiviral role, autophagy can also be exploited by viruses to promote their own multiplication. Viruses commonly harness autophagy to degrade some host defense components, thereby benefiting their replication. One such example is the utilization of autophagy by viruses to counteract the antiviral response mediated by RNA silencing, a highly effective strategy employed by hosts to suppress viral infection [76]. In this context, certain viral VSRs target key components involved in RNA silencing, such as AGOs and plant endogenous suppressor of gene silencing 3 (SGS3) [77], for autophagic degradation. For instance, the P0 of poleroviruses, which acts as a VSR, directly interacts with AGO1 and promotes its degradation through autophagy [78]. Intriguingly, the autophagic degradation of AGO1 relied on its ubiquitylation, mediated by SCF-type E3 ligases. The conserved F-box motif in P0 interacts with SKP1, facilitating the recruitment of SCF-type E3 ligases for the ubiquitylation of AGO1 [79,80,81]. Tong et al. (2021) reported that CMV-induced VISP1 can mediate the degradation of suppressor of gene silencing 3/RNA-dependent RNA polymerase 6 (SGS3/RDR6) bodies through autophagy, thus restraining the antiviral RNA silencing to promote virus infection [82]. Similarly, TYLCCNB, a geminivirus, upregulates a calmodulin-like protein, namely NbCAM, to suppress RNA silencing and promote viral infection by degrading SGS3 via the autophagy pathway [83]. Beside this, some viral VSRs, such as the viral protein genome-linked (VPg) of potyviruses and the matrix protein (M) of rice stripe mosaic virus (RSMV), counteract RNA-silencing-mediated antiviral responses by directly promoting SGS3’s degradation. In these cases, SGS3 undergoes degradation through both the UPS and autophagy pathways [84,85]. Apart from this, autophagy has been reported to contribute to viral cell-to-cell movement. Fu et al. (2018) reported that RSV interferes with the S-acylation of the remorin protein in *N. benthamiana* (NbREM1) and induces its autophagic degradation to facilitate virus infection [86]. NbREM1 regulates callose deposition at the neck region of plasmodesmata, limiting virus cell-to-cell trafficking. The RSV-encoded movement protein, NSvc4, disrupts NbREM1 S-acylation by binding to its C-terminal domain. The S-acylation-deficient NbREM1 triggers autophagy-mediated degradation, thereby benefiting virus infection [86]. In the above cases, viruses exploit autophagy to target host-defense-related factors for degradation. However, this is not the sole way in which viruses exploit autophagy. Li et al. (2020) reported that TuMV co-opts the NBR1-ATG8f-TIP1 module to facilitate virus replication and virion accumulation within the vacuole [87]. They also observed that the βC1 protein of CLCuMuB hijacks the host autophagic receptor NbNBR1 to form cytoplasmic granules, thereby protecting itself from UPS-mediated degradation and promoting viral infection [37]. Hafrén et al. reported that CaMV and TuMV utilize NBR1-independent autophagy to enhance the survival of infected plants and serve viruses by prolonging the timespan for viral proliferation [61,62].

## 4. Conclusions

This review provides a comprehensive summary of the current knowledge regarding the interplay between viral infection and protein degradation mediated by the plant UPS and autophagy. It is now clear that both autophagy and the UPS act as double-edged swords in the ongoing battle between plants and viruses. However, existing studies have primarily focused on specific viral or plant proteins, overlooking the crosstalk within and between the pathways of the UPS and autophagy. For example, NBR1-mediated selective autophagy participates in the degradation of the CaMV P4 protein, while NBR1-independent bulk autophagy prolongs plant’s timespan for viral proliferation [61]. A similar scenario is observed in the case of TuMV, where the Beclin-mediated autophagy pathway degrades the TuMV NIb protein [64], but the virus can hijack the NBR1-mediated autophagy pathway to enhance virus replication and virion accumulation within the vacuole [87]. Another example involves the βC1 protein encoded by geminiviruses, which can be degraded by both cellular autophagy and the UPS pathway [36,68,70]. Conversely, the βC1 protein can exploit cellular autophagy to degrade the host’s SGS3 protein and also subvert the degradation of the jasmonate ZIM-domain (JAZ) proteins through the SCF complex, thereby creating a cellular environment conducive to viral infection [45,83]. Understanding how viruses balance multiple distinct cellular UPS/autophagy pathways to achieve optimal intracellular survival is crucial. Is there a mechanism that enables viruses to activate the UPS/autophagy pathways that benefit their own survival while inhibiting antiviral-related UPS/autophagy pathways? Another crucial aspect often overlooked is how specific cell types respond to viral infection. Existing research has predominantly focused on leaf tissue, leaving a significant knowledge gap regarding UPS-/autophagy-mediated protein homeostasis in other cell types, particularly meristems, during viral infections. It can be assumed that, in mature cells undergoing PCD, the UPS/autophagy serves to recycle various cellular components, whereas in meristem cells, which do not undergo PCD, the UPS/autophagy tends to become relatively “dormant”. Notably, the meristem central and peripheral zones are known to be free from viral intrusion [88]. Could this be related to the relatively “dormant” state of UPS/autophagy in these cells? By thoroughly analyzing the relationship between UPS-/autophagy-mediated protein homeostasis and viral infection as a whole, while also investigating cell-type-specific responses to viral infection, we can acquire a comprehensive understanding of the pivotal role played by the UPS/autophagy in viral invasion.

## Data Availability

Not applicable.
